# Effects and safety of propofol intravenous anesthesia in transvaginal oocyte retrieval on outcomes of *in vitro* fertilization and embryo transplantation

**DOI:** 10.3389/fendo.2024.1497948

**Published:** 2024-12-16

**Authors:** Xiao-Ming Liu, Fan Zhang, Xiao-Sheng Lu, Hai-Tao Xi, Jun-Zhao Zhao

**Affiliations:** Reproductive Medicine Centre, Department of Obstetrics and Gynecology, the Second Affiliated Hospital of Wenzhou Medical University, Wenzhou, China

**Keywords:** oocyte retrieval, *in vitro* fertilization, pregnancy rate, embryo quality, propofol

## Abstract

**Purpose:**

Propofol, a widely utilized anesthetic, is employed to alleviate pain and anxiety in outpatient oocyte retrieval procedures. However, its potential impact and safety profile in the context of *in vitro* fertilization and embryo transfer (IVF-ET) remain unclear.

**Methods:**

This retrospective study enrolled 1187 patients undergoing IVF-ET, and divided into two groups depending on whether they received propofol (propofol group, n=140) or not (control group, n=1047) for anesthesia during oocyte retrieval.

**Results:**

The baseline characteristics were comparable between the groups. Compared with control group, the number of oocytes retrieved in propofol group was more (*p*=0.012), while both the estradiol (E2) level on the trigger day and the pre-ovulatory follicle count were higher in propofol group ((*p*<0.01). Additionally, the rate of preterm delivery was significantly higher in the propofol group (p<0.001). To further analyze the effect of propofol on the oocyte retrieval rate, patients were divided into three subgroups depending on the pre-ovulatory follicle count (≤10, 11–20, and >20) to eliminate the influence of inconsistency in the estimation of the pre-ovulatory follicle count between the two groups. Analysis revealed that the use of propofol during oocyte retrieval was particularly advantageous in the subgroup with a pre-ovulatory follicle count of 11–20, yielding a higher oocyte retrieval rate (p<0.001).

**Conclusion:**

The use of propofol in oocyte retrieval did not adversely affect fertilization, embryo quality, or clinical outcomes. Moreover, it was found to increase the oocyte retrieval rate among patients with an estimated pre-ovulatory follicle count of 11–20. These findings offer valuable evidence supporting the clinical application of propofol in oocyte retrieval procedures.

## Introduction

Infertility is the inability to conceive within 1 year of unprotected intercourse and has been identified as a public health priority (1). In recent years, the incidence of infertility has increased annually, and the prevalence of infertility is about 25% among couples of reproductive age in China (2). As one of the leading treatments for infertility, *in vitro* fertilization and embryo transfer (IVF-ET) is a major assisted reproductive technique with a high success rate (3). During the process of IVF-ET, the most fearful part for patients is oocyte retrieval. To reduce the pain and fear of patients, anesthesia is applied gradually for oocyte retrieval. However, the safety of anesthesia on IVF-ET outcomes is yet unclear.

Transvaginal ultrasound-guided oocyte retrieval is a painful assisted reproductive technology procedure. A small number of patients are in severe pain due to anatomic changes in the pelvis, such as adenomyosis and chronic pelvic inflammation (4). Hitherto date, both general and regional anesthetics, including paracervicals, spinals, and epidurals have been used, and various methods of conscious sedation and analgesia have been attempted to reduce the pain of patients (5). Whenever favorable analgesia with sedation and rapid recovery is desired, propofol and remifentanil are administered in ambulatory settings due to their pharmacokinetic profile (6, 7). Propofol (2,6-diisopropylphenol, Diprivan, ICI Pharmaceuticals, Manchester, UK) is widely used either as an adjunct in general anesthesia or as a sole anesthetic agent by continuous intravenous administration and intermittent bolus injections for minor surgical interventions. For several years, this anesthetic was used for painless oocyte retrieval in IVF (8). Some studies have shown that propofol does not affect the postoperative levels of female sex hormones (serum estradiol and follicle-stimulating hormone [FSH]) after gynecologic surgery (9). Nonetheless, the potential impact of propofol used in oocyte retrieval on oocyte fertilization, embryo quality, and clinical pregnancy also need to be explored further. The results of the studies on the concentrations of propofol in follicular fluid during oocyte retrieval in women showed that the anesthetic accumulated in the follicular fluid in a dose- and time-dependent manner, which might have potential adverse effects on the follicular quality (10). A prolonged duration of anesthesia (>30 min) might seem to decrease implantation and clinical pregnancy rates (7, 11). Some studies showed that propofol has no effect on polar body extrusion in oocyte maturation, pronucleus formation, and embryo development of mice (12). However, a recent study showed that the embryo number and quality, and pup count of rats decreased with an increasing time of propofol administration (13). Another study showed that propofol does not affect the fertilization rate compared to an anesthetic ketamine (11); however, the data of patients without anesthesia were not included and compared to the propofol data in the study. Therefore, the effect of propofol on fertilization, embryo quality, and offspring is still inconclusive and needs to be studied further.

Therefore, the present study was undertaken to explore the effects and safety of propofol on the oocyte retrieval rate, embryo quality, and pregnancy outcomes.

## Methods

### Reasons for selecting retrospective analysis in this study

The use of a retrospective design in the study investigating the effects of propofol on *in vitro* fertilization and embryo transfer (IVF-ET) has several justifications, particularly when compared to prospective or randomized approaches. Here are the key reasons:

Ethical Considerations: Retrospective studies are often chosen when there are ethical concerns that prevent the use of a traditional experimental design. In the context of IVF-ET, it may not be ethical to withhold anesthesia from a control group, making a retrospective design more suitable.

Efficiency in Time and Budget: Retrospective studies are more efficient in terms of time and budget. They require fewer subjects and utilize pre-existing secondary research data, which is cost-effective and less time-consuming compared to the extensive planning and execution required for prospective or randomized studies.

Studying Rare or Unusual Exposures: Retrospective cohort studies are particularly useful when studying rare or unusual exposures, as well as diseases with a long latency or incubation period. This is relevant in IVF-ET where the use of propofol may be less common or have long-term effects that are not yet fully understood.

Relatively Inexpensive and Quick: The use of previously collected and stored records in a database means that retrospective cohort studies are relatively inexpensive and quick to perform. This is an advantage over prospective studies, which require significant resources for data collection and follow-up.

Sequence of Risk and Outcome Factors: Both retrospective and prospective cohort studies allow for the recording of exposure to risk factors before the outcome occurs, which is crucial for evaluating the sequence of risk and outcome factors.

However, it is important to acknowledge the limitations of retrospective studies, such as the risk of research biases, including recall bias and observer bias due to reliance on memory and self-reported data. Additionally, retrospective studies cannot establish causality, leading to lower internal and external validity. Despite these limitations, retrospective studies can provide valuable preliminary data that can inform the design of larger, more rigorous prospective or randomized trials.

### Subjects

This retrospective study consecutively enrolled 1187 patients who were treated with IVF-ET cycles between June 2016 and 2017 at the Reproductive Center of Second Affiliated Hospital of Wenzhou Medical University as the study subjects. Patient-related data were retrieved from the hospital’s electronic database system.

The inclusion criteria were as follows: (1) all candidates were aged 21–46-years-old and met the IVF-ET indications; (2) all candidates undergoing IVF-ET for the first time; (3) all candidates were subjected to the standardized long agonist protocol; (4) the number of oocytes collected was >4.

The exclusion criteria were as follows: (1) endometrial adhesion, submucosal myoma, or uterine diameters >65 mm: these conditions can significantly affect the uterine environment and the success of IVF-ET procedures. Including patients with these conditions could introduce confounding variables that might skew the results, making it difficult to attribute outcomes to propofol anesthesia alone; (2) obvious infection after oocyte retrieval: infections can have serious implications for patient health and can also affect the success of IVF-ET; (3) history of cardiopulmonary disease, hypertension, opioids, and benzodiazepines, which can have systemic effects on the body, potentially influencing the outcomes of IVF-ET (For example, cardiopulmonary disease can affect oxygenation and blood flow, which are critical for embryo development); (4) complications with malignant tumors or other systemic diseases, such as an active stage of systemic lupus erythematosus, that were not suitable for pregnancy.

### Study design

**Figure 1:**
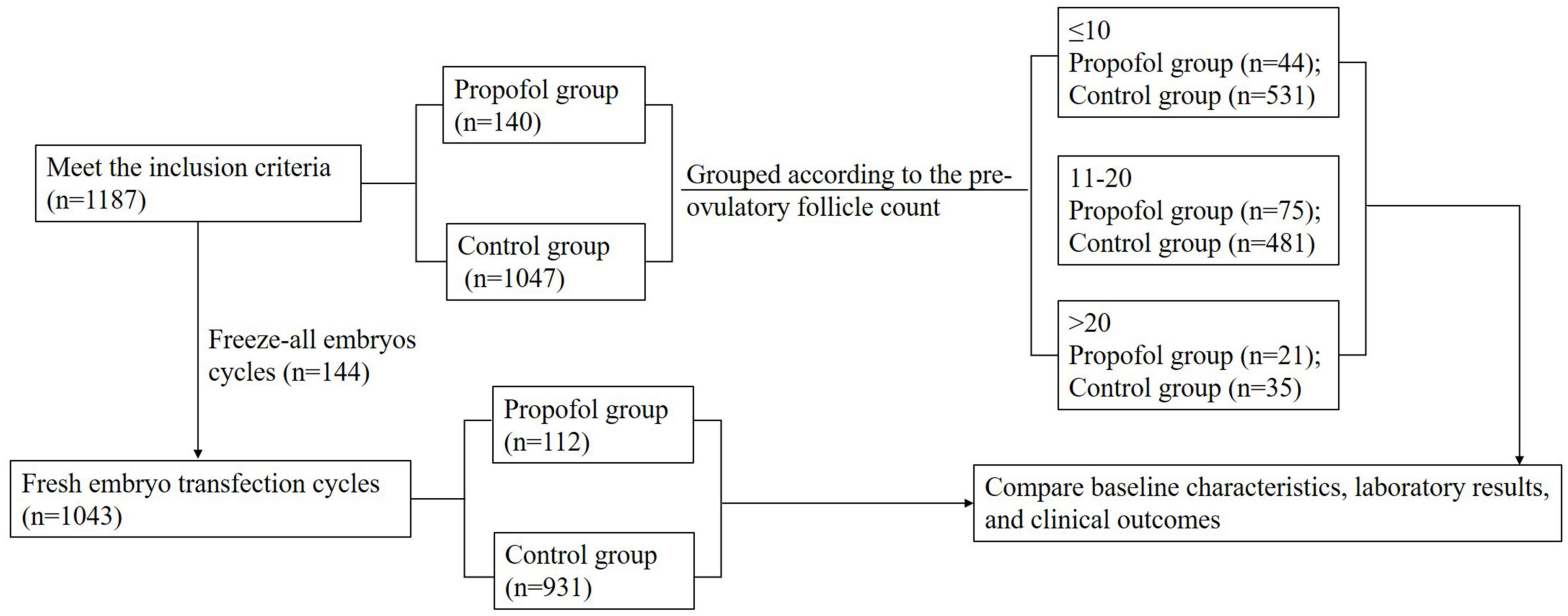
Flowchart. A total of 1187 cycles were consecutively enrolled in our single reproductive center during June 2016–2017. Among these, 1043 cycles underwent fresh embryo transfection. Patients who employed propofol as an anesthetic for oocyte retrieval were consecutively enrolled in the propofol group (n=140), and the others comprised the control group (n=1047). Then, each group was divided into three subgroups according to the pre-ovulatory follicle count (≤10, 11–20, and >20). The baseline information, laboratory data, and clinical outcomes were analyzed between each subgroup.

Oocyte retrieval with an anesthetic is not a routine operation during IVF-ET in China. The pain caused by oocyte retrieval is tolerable by most patients. However, patients who could not stand the pain and met the criteria of anesthetic surgery could select anesthetics during oocyte retrieval. Therefore, the patients who were administered propofol as an anesthetic during oocyte retrieval were consecutively enrolled in the current study as the propofol group (n=140). The other patients enrolled during the same period who met the inclusion criteria comprised the control group (n=1047). All oocyte retrieval procedures were performed by the same gynecologist. Oocyte retrieval rate, metaphase II (MII) oocyte rate, two pronuclear (2PN) rate, cleavage rate, high-quality embryo rate, and frozen embryo rate were analyzed. Among the 1187 patients, 1043 underwent fresh embryo transfer (propofol group n=112, control group n=931); then, the biochemical pregnancy, clinical pregnancy rate, early spontaneous abortion rate, ectopic pregnancy rate, multiplets pregnancy rate, preterm delivery rate, neonate weight, and sex ratio were analyzed between the two groups (**Figure 1**).

In order to further analyze the correlation between the oocyte retrieval rate and the oocyte retrieval operation with or without anesthetics, the patients were divided into three subgroups depending on the pre-ovulatory follicle count (≤10, 11–20, and >20) to eliminate the influence of inconsistent estimated pre-ovulatory follicle count between the propofol and control groups (**Figure 1**).

### Superovulation program and embryo culture

All patients were subjected to a long-term protocol of pituitary downregulation with gonadotrophin-releasing hormone (GnRH) agonist for controlled ovarian hyperstimulation (COH) as described previously (14).

Patients who underwent oocyte retrieval with anesthesia were deprived of food and water for >10 h. Then, intravenous access was established and transferred into the operation room after bladder voiding. Propofol was administered as the anesthetic agent for the oocyte retrieval procedure. Dosing was tailored to each patient’s weight, age, and overall health status, with a standard dosage ranging from 2 to 3 milligrams per kilogram of body weight. The operation was efficiently performed and completed within a 20-minute timeframe. The vital signs were closely monitored during the operation, including the blood pressure (BP), mean arterial pressure (MAP), heart rate (HR), and oxygen saturation (SpO2), and oxygen was administered at 2-3 L/min. Postoperative monitoring included electrocardiogram (ECG), non-invasive blood pressure (NIBP), and SpO2 every 15 min, along with pain (VAS scores) and nausea/vomiting (PONV) evaluation.

The embryos were cultured as described previously, and the embryos were scored according to the 2011 Istanbul Consensus (15, 16). Briefly, fertilization was observed 20 h after insemination based on the appearance of 2PN. The embryos were cultured in G-1 plus and G-2 plus medium (Vitrolife Co., Ltd, Australia) at 37°C in a humidified atmosphere with 6% CO_2_. A D3 embryo with 7–9 blastomere cells of an A or B grade was considered a high-quality D3 embryo (15).

### ET and luteal phase support

ET was performed under the guidance of transabdominal ultrasound. The starting time of luteal phase support depended on the serum P4 level on the day of hCG trigger, as described previously (17). Briefly, when P was <1.2 ng/mL, luteal support was initiated 1 day after oocyte retrieval. When P was ≥1.2 ng/mL, luteal support was initiated 2 days after ovulation. If P was ≥1.5 ng/mL, ET was canceled. Crinone gel and dydrogesterone tablets were used for luteal phase support until days 13-14 after ET. In the case of pregnancy, luteal support was continued until weeks 10–12 of pregnancy.

### Outcome assessment

The present study aimed to analyze the laboratory data, including the oocyte retrieval rate, MII oocyte rate, 2PN rate, cleavage rate, high-quality embryo rate, frozen embryo rate, and the clinical outcomes, which included biochemical pregnancy, clinical pregnancy rate, early spontaneous abortion rate, ectopic pregnancy rate, multiplets pregnancy rate, preterm delivery rate, and neonate weight.

We also defined the laboratory and pregnancy outcomes in this study according to “CSRM consensus on the key indicators for quality control in IVF laboratory” (18).

1. Oocyte retrieval rate: number of oocytes retrieved/pre-ovulatory follicle count (follicle diameter ≥12 mm was counted on the hCG injection day)×100%.
2. MII oocyte rate: number of MII oocytes/number of oocytes retrieved×100%.
3. 2PN rate: number of 2PN fertilized oocytes/number of MII oocytes×100%.
4. Cleavage rate: number of cleaved embryos/number of 2PN fertilized oocytes×100%.
5. High-quality embryo rate: number of high-quality D3 cleaved−embryos/number of cleavage embryos×100%. Note, a D3 embryo with 7–9 blastomere cells of an A or B grade was considered a high-quality D3 embryo.
6. Frozen embryo rate: number of frozen embryos/(total number of embryos cultured−total number of embryos transferred)×100%.
7. Biochemical pregnancy rate: biochemical pregnancy cycle number/fresh transfer cycle number×100%. The biochemical pregnancy was diagnosed when serum β-hCG level was >25 IU/L on the 14^th^ day after embryo transfer.
8. Clinical pregnancy rate: clinical pregnancy cycle number/fresh transfer cycle number×100%. Clinical pregnancy was diagnosed when ≥1 pregnancy sacs were observed on ultrasound. The rate included normal intrauterine pregnancy, ectopic pregnancy, and heterotopic pregnancy.
9. Early spontaneous abortion rate: number of spontaneous abortion cycles within 12 weeks of pregnancy/number of clinical pregnancy cycles×100%.
10. Ectopic pregnancy rate: number of ectopic pregnancy cycles/number of clinical pregnancy cycles×100%.
11. Multiplets pregnancy rate: multiple pregnancy cycles/number of clinical pregnancy cycles×100%.
12. Live birth rate: number of live births/transfer cycles×100%.
13. Preterm delivery rate: number of live births before 37 weeks/number of live births×100%.

### Statistical analysis

The data were analyzed using the SPSS software (Version 17.0; SPSS, Chicago, IL, USA). Student’s *t*-test was used to analyze the continuous variables, and the chi-square test was used for non-continuous variables. Before data analysis, a normality test was conducted. For percentage data that deviates from a normal distribution, such as multiple pregnancy rates and preterm birth rates, an arcsine transformation was performed. After the transformation, the data conformed to a normal distribution, and the t-test was continued for analysis. All data are presented as mean ± standard deviation (SD), and *p*<0.05 was considered statistically significant.

## Results

### General characteristics

In this study, we analyzed 1187 IVF-ET cycles in patients who, for the first time, underwent the assisted reproductive technology (ART) treatment (**Figure 1**). We compared the patients’ clinical outcomes, including the baseline information, the clinical parameters, the laboratory data, and the clinical pregnancy outcomes. The baseline information, including age, infertility duration, body mass index (BMI), basal FSH, LH, E2, P, and the antral follicle count (AFC) were similar between the two groups (**Table 1**).

**Table 1:** General characteristics of patients at baseline.

	Propofol group (n=140)	Control group (n=1047)	*p-*value
Maternal age (years)	30.6 (4.4)	32.1 (4.6)	0.439
Infertility duration (years)	3 (2.4)	3.1 (1.9)	0.12
Maternal BMI (kg/m^2^)	20.9 (2.9)	21.6 (2.6)	0.067
Basal FSH (mIU/mL)	6.7 (2.6)	6.7 (2.2)	0.065
Basal LH (mIU/mL)	4.2 (1.8)	4.3 (1.9)	0.263
Basic E2 level (pg/mL)	50.6 (18.4)	51.9 (16.6)	0.124
Progesterone (ng/mL)	0.51 (0.27)	0.51 (0.26)	0.339
AFC	13.3 (5.8)	11.3 (5.8)	0.841

Data are presented as mean (SD). No statistically significant differences were observed between the two groups. BMI: body mass index; FSH: follicle-stimulating hormone; LH: luteinizing hormone; E2: estradiol; AFC: antral follicle count.

### Clinical parameters

All patients received a long-term protocol, and the clinical parameters are shown in **Table 2**. The level of E2 (2403.7 ± 1276.6 pg/mL *vs*. 2092.0 ± 1015.9 pg/mL, *p*=0.001) and the pre-ovulatory follicle count (14.2 ± 6.8 *vs*. 11.1 ± 4.7, *p*<0.001) was significantly higher in the propofol group than in control group. On the other hand, the total dose of Gn and days of Gn were similar between the two groups.

**Table 2:** Clinical parameters.

	Propofol group (n=140)	Control group (n=1047)	*p*-value
Total does of Gn (U)	2163.8 (794.0)	2490.6 (927.0)	0.154
Gn duration (days)	11.6 (2.2)	11.6 (2.6)	0.135
E2 on the trigger day (pg/mL)	2403.7 (1276.6)	2092.0 (1015.9)	0.001
Pre-ovulatory follicle count	14.2 (6.8)	11.1 (4.7)	0

Data are presented as mean (SD). Gn: gonadotrophin; E2: estradiol.

### *In vitro* fertilization outcome parameters

The laboratory results are summarized in **Table 3**. The number of oocytes retrieved, number of MII oocytes, number of 2PN oocytes, number of D3 high-quality embryos, and the number of frozen embryos of the propofol group were significantly higher in the propofol group than in the control group (*p*<0.05). Conversely, no significant difference was detected in the MII oocyte rate, 2PN rate, number of cleavage oocytes, cleavage rate, D3 high-quality embryo rate, and frozen embryo rate between the two groups. However, the oocyte retrieval rate was significantly higher in the control group (109 ± 36.4%) than in the propofol group (107 ± 41.1%, *p*=0.007).

**Table 3:** *In vitro* fertilization laboratory data.

	Propofol group (n=140)	Control group (n=1047)	*p*-value
No. of oocytes retrieved	13.6 (5.2)	11.4 (4.3)	0.012
Oocyte retrieval rate (%)	107 (41.1)	109 (36.4)	0.007
No. of MII oocytes	11.9 (4.9)	9.7 (4.0)	0.007
MII oocyte rate (%)	87.7 (13.4)	85.4 (15)	0.081
No. of 2PN oocytes	9.5 (4.1)	7.8 (3.5)	0.038
2PN rate (%)	80.2 (15.4)	80.4 (16.5)	0.297
No. of cleavage embryos	9.2 (4.0)	7.6 (3.5)	0.139
Cleavage rate (%)	97.5 (6.1)	97.8 (6.6)	0.612
No. of D3 high-quality embryos	3.7 (2.6)	2.7 (2.3)	0.004
High-quality embryo rate (%)	41.2 (25.6)	34.2 (24.7)	0.283
No. of frozen embryos	4.6 (3.3)	3.2 (2.8)	0.004
Frozen embryo rate (%)	60.4 (33.2)	51.7 (34.8)	0.299

Data are presented as mean (SD). No.: number; MII: metaphase II; 2PN: two pronucleus.

### Pregnancy outcome parameters

A total of 1043 patients underwent fresh embryo transfer (propofol group n=112, control group n=931). As shown in **Table 4**, the propofol and control groups had similar biochemical pregnancy rates, clinical pregnancy rates, early spontaneous abortion rates, ectopic pregnancy rates, multiplets pregnancy rates, live birth rates, neonate weight, and male to female ratio. However, a statistically significant difference was detected in the preterm delivery rates between the propofol group [9/65 (13.8%)] and the control group [12/591 (2%), *p<0.001*], and twin pregnancy was the main cause of preterm delivery [7/65 (10.8%) *vs*. 6/591 (1%), *p<0.001*].

**Table 4:** *In vitro* fertilization-embryo transfer pregnancy outcomes.

	Propofol group (n=112)	Control group (n=931)	*p*-value
Biochemical pregnancy rate	8/112 (7.1%)	55/931 (5.9%)	0.604
Clinical pregnancy rate	60/112 (53.6%)	534/931 (57.4%)	0.445
Early spontaneous abortion rate	9/60 (15%)	61/534 (11.4%)	0.415
Ectopic pregnancy rate	0/60 (0%)	5/931(0.5%)	0.569
Multiplets pregnancy rate	18/60 (30%)	133/534 (24.9%)	0.39
Live birth rate	65/112 (58.0%)	591/931 (63.5%)	0.26
Preterm delivery rate	9/65 (13.8%)	12/591 (2.0%)	0
Preterm delivery rate of twin pregnancy	7/65 (10.8%)	6/591 (1.0%)	0
Neonate weight (g)	2826.0 (673.0)	2922.0 (657.0)	0.281
Male to female ratio	36/29 (1.34:1)	289/302 (0.96:1)	0.321

Data are presented as mean (SD) or n (%).

### Oocyte retrieval under painless operation is valuable to obtain oocytes

To investigate the relationship between the oocyte retrieval rate and the use of anesthesia during the procedure, patients were stratified into three distinct subgroups based on the number of pre-ovulatory follicles. This stratification aimed to mitigate the impact of potential disparities in the estimation of pre-ovulatory follicle counts between the propofol and control groups. The three subgroups were as follows: pre-ovulatory follicle count ≤10 group (Group 1; painless group, n=44; control group, n=531), pre-ovulatory follicle count 10–20 group (Group 2; painless group, n=75; control group, n=481), and pre-ovulatory follicle count >20 group (Group 3; painless group, n=21; control group, n=35). Pre-ovulatory follicle is a non-invasive diagnostic tool that assists clinicians in estimating the quantity of oocytes that can be harvested and the likelihood of success in an IVF cycle. The thresholds established signify varying degrees of ovarian reserve.

The baseline information, clinical parameters, and laboratory data were compared among the subgroups. The baseline information and clinical parameters are summarized in supplementary **Supplementary Tables S1**, **S2**. Furthermore, no significant differences were observed in age, infertility duration, basal FSH, LH, E2, and P, AFC, days of Gn, total dose of Gn, E2 on the trigger day, and pre-ovulatory follicle count between Groups 1 and 2, whereas the pre-ovulatory follicle count was higher in the propofol group than in the control group in Group 3 (26.1 ± 5.8 *vs*. 23.1± 2.2, *P*=0.002).

The IVF outcome parameters are listed in **Table 5**; the results in the propofol group were similar to those of the control group in Group 1, while in Group 2, the number of oocytes retrieved (14.8 ± 4.9 *vs*. 14.1 ± 3.5, *p*=0.001) and the oocyte retrieval rate (103.4 ± 36% *vs*. 97.9 ± 23.1%, *p*<0.001) were significantly higher in the propofol group than in the control group. Moreover, the number of MII oocytes was higher in the propofol group than in the control group (*p*=0.01). The MII oocyte rates, number of 2PN oocytes, 2PN oocyte rates, number of cleavage embryos, number of D3 high-quality embryos, high-quality embryo rates, number of frozen embryos, and the frozen embryo rates were higher in the propofol group, but the differences were not statistically significant in Groups 1 and 2. In Group 3, the pre-ovulatory follicle count was higher in the propofol group than in the control group (26.1 ± 5.8 *vs*. 23.1 ± 2.2, *p*=0.002); however, the number of oocytes retrieved was similar between the two groups (*p*=0.53).

**Table 5:** *In vitro* fertilization laboratory data.

	Group 1 (≤10)			Group 2 (11–20)			Group 3 (>20)		
	Propofol group	Control group	*p-*	Propofol group	Control group	*p-*	Propofol group	Control group	*p-*value
	(n=44)	(n=531)	value	(n=75)	(n=481)	value	(n=21)	(n=35)	
Pre-ovulatory follicle count	7.4 (1.9)	7.3 (1.8)	0.388	14.7 (2.8)	14.6 (2.5)	0.231	26.1 (5.8)	23.1 (2.2)	0.002
No. of oocytes retrieved	9.5 (3.1)	8.7 (3.0)	0.875	14.8 (4.9)	14.1 (3.5)	0.001	17.4 (4.4)	16.5 (3.7)	0.53
Oocytes retrieved rate (%)	131.7 (40.7)	122.0 (41.8)	0.641	103.4 (36.0)	97.9 (23.1)	0	68.3 (20.1)	72.0 (17.1)	0.567
No. of MII oocytes	8.5 (2.9)	7.2 (2.8)	0.485	13.0 (4.5)	12.1 (3.3)	0.01	14.9 (5.5)	14.3 (3.5)	0.006
MII oocytes rate (%)	89.0 (14.3)	84.0 (16.4)	0.261	87.9 (11.3)	86.7 (13.4)	0.087	84.0 (17.8)	87.0 (11.3)	0.029
No. of 2PN oocytes	7.1 (2.8)	5.8 (2.5)	0.259	10.2 (3.8)	9.8 (3.3)	0.112	10.8 (5.0)	11.2 (3.5)	0.021
2PN rate (%)	83.0 (15.9)	80.5 (18.2)	0.219	81.0 (14.5)	80.5 (14.6)	0.592	71.9 (15.2)	77.8 (14.6)	0.49
No. of cleavages	6.9 (2.7)	5.7 (2.4)	0.298	10.2 (3.8)	9.5 (3.3)	0.112	10.5 (5.0)	10.8 (3.4)	0.042
Cleavage rate (%)	97.5 (6.0)	98.3 (7.0)	0.44	97.4 (5.3)	97.4 (6.2)	0.794	97.7 (8.9)	96.8 (5.0)	0.984
No. of D3 high-quality embryo	3.0 (1.9)	1.8 (1.7)	0.092	4.0 (2.8)	3.5 (2.5)	0.058	4.0 (3.3)	4.0 (2.9)	0.294
High-quality embryo rate (%)	44.2 (27.2)	31.8 (26.5)	0.691	40.3 (24.1)	36.7 (22.6)	0.397	38.1 (28.1)	36.5 (19.9)	0.025
No. of frozen embryo	3.3 (2.5)	2.0 (2.0)	0.07	5.1 (3.3)	4.4 (3.0)	0.176	5.9 (4.0)	4.8 (3.1)	0.227
Frozen embryo rate (%)	59.1 (36.4)	49.0 (38.1)	0.356	59.4 (29.4)	54.6 (31.8)	0.924	67.0 (39.5)	50.9 (21.2)	0.187

Data are presented as mean (SD). No.: number; MII: metaphase II; 2PN: two pronucleus.

## Discussion

Herein, we aimed to evaluate the effect of propofol anesthetic used in IVF-ET on IVF outcomes, including oocyte retrieval parameters and clinical success. The results showed that oocyte retrieval under anesthesia with propofol in IVF-ET had no negative effects on the fertilization rate, cleavage rate, embryo quality, frozen embryo rate, clinical pregnancy rate, live birth, neonate weight, and sex ratio, and no statistically significant difference was detected between the propofol group and control group. Furthermore, the oocyte retrieval operation under anesthesia could get more oocytes when patients had a pre-ovulatory follicle count of 11–20. The current study revealed that the outcome for oocyte retrieval under anesthesia with propofol was safe with respect to IVF-ET parameter; thus, it might be a safe and helpful choice for patients to reduce the pain and fear while the embryo quality and clinical pregnancy may not be influenced.

For several years, propofol has been used as a kind of anesthesia in transvaginal oocyte retrieval for IVF, allowing a completely painless puncture on an outpatient basis. Previous studies have suggested that propofol may accumulate in follicular fluid (10), and dose-and time-dependent toxic effects of propofol on fertilization rates were reported in mice (19). However, no detrimental effects of propofol on fertilization and the quality of embryos were detected in humans (20). In the present study, the fertilization rate was similar between the propofol and the control groups, consistent with the previous study (11, 21). Unexpectedly, the number of oocytes retrieved, number of normally fertilized oocytes, number of D3 high-quality embryos, and number of frozen embryos in the propofol group were all higher than that in the control group. It seems that propofol used in oocyte retrieval was beneficial and harmless to IVF-ET, while a previous study showed no difference in the embryo quality between patients who received propofol or other anesthesia (21). However, the cited study’s limitation is that it did not include or analyze patients who did not receive anesthesia. Considering that the control group was not subjected to any form of anesthesia, it would be advantageous to compare our findings with those from studies that have assessed different anesthetic agents. Consequently, our research would provide a more comprehensive context for understanding the relative safety of propofol.

One of the important aspects of this study that should be discussed is the relationship between age and oocyte retrieval rate. In the present study, the age in the propofol group was younger than that in the control group, and the number of AFC in the propofol group was more than in the control group, although not significantly. However, the level of E2 on the trigger day and the pre-ovulatory follicle count was significantly higher in the propofol group compared with the control group. Previous studies showed that age and AFC are related to fertility in domestic animals, younger with higher AFC (22). Furthermore, AFC was also correlated with oocyte count and the number of fresh and frozen embryos in the patients between 20-32 years old (23), which was consistent with the present study. However, the oocytes retrieved rate in the control group was higher than in the propofol group. In order to further analyze the relationship between the oocyte retrieval rate and oocyte retrieval operation with or without propofol, we divided the groups into three subgroups depending on the pre-ovulatory follicle count. The data showed that in Group 2, the number of oocytes retrieved, oocyte retrieval rate, and number of MII oocytes, were significantly higher in the propofol group compared to the control group; meanwhile, in Group 3, the propofol group had more mature oocytes and high-quality embryos than in the control group. The results indicated that propofol used during IVF-ET is beneficial for patients.

Another important aspect of this study that should be discussed is the effect of propofol on IVF-ET pregnancy outcomes. A non-significant difference in the biochemical pregnancy rate, the clinical pregnancy rate, early spontaneous abortion rate, multiplets pregnancy rate, ectopic pregnancy rate, and live birth was observed in the present study. However, the preterm delivery rate was significantly higher in the propofol group than in the control group, and the preterm delivery rate of twin pregnancy was also significantly higher in the propofol group. Twin pregnancies are at increased risk of preterm delivery and constitute at least 10% of cases of preterm delivery (24). The preterm birth risk was relatively low for women in their late 30s; however, a twin pregnancy is associated with an increased risk for most adverse perinatal outcomes (25). The patients in the propofol group were younger than those in the control group, and they may prefer to have twin pregnancies at one time and select two embryos for transfer; however, the risk of twin pregnancy could not be ignored. The results hint that fresh Single-blastocyst-transfer (SBT) may offer patients efficiency and security, as SBT can prevent high incidences of complications such as multiple pregnancies and ovarian hyperstimulation syndrome (26). On the other hand, some studies suggests that propofol could be associated with an elevated risk of preterm birth, possibly exerting its influence on pregnancy outcomes through several pathways (27). These include its possible effects on fetal organ development and alterations in maternal hemodynamics (28). Consequently, additional studies are imperative to elucidate the precise impact of propofol on preterm birth rates and to uncover the mechanisms at play.

However, it is important to acknowledge the limitations of retrospective studies, such as the risk of research biases, including recall bias and observer bias due to reliance on memory and self-reported data. Additionally, retrospective studies cannot establish causality, leading to lower internal and external validity. Despite these limitations, this retrospective study can provide valuable preliminary data that can inform the design of larger, more rigorous prospective or randomized trials.

Owing to the constraints imposed by a limited sample size and potential selection bias inherent in a single-center retrospective study, future investigative efforts should prioritize multi-center collaborations to broaden and diversify the dataset. It is also imperative to incorporate regional patient demographics to facilitate a more nuanced and thorough assessment of propofol’s safety during the oocyte retrieval process. Furthermore, the long-term safety for offspring must not be overlooked, necessitating additional clinical follow-up studies to thoroughly address this critical aspect.

## Conclusion

The findings of this study indicate that the use of propofol during oocyte retrieval significantly enhanced the oocyte retrieval rate among patients with an estimated pre-ovulatory follicle count between 11 and 20. Importantly, propofol did not adversely affect key reproductive outcomes, including fertilization rate, embryo cleavage rate, embryo quality, frozen embryo rate, clinical pregnancy rate, biochemical pregnancy rate, early spontaneous abortion rate, multiplets pregnancy rate, ectopic pregnancy rate, live birth rate, and sex ratio. However, a notable increase in the preterm delivery rate was observed in the propofol group compared to the control group, suggesting that the potential risks associated with twin pregnancies should not be overlooked.

## Data Availability

The original contributions presented in the study are included in the article/**Supplementary Material**. Further inquiries can be directed to the corresponding author.
