# Supplementary material for: Effects and safety of propofol intravenous anesthesia in transvaginal oocyte retrieval on outcomes of in vitro fertilization and embryo transplantation
Source: Front Endocrinol (Lausanne). 2024 Dec 16;15:1497948. doi: 10.3389/fendo.2024.1497948 (PMC11686430; doi:10.3389/fendo.2024.1497948)
Supplement: Supplementary file 1 [file Table1.docx]

**Supporting Information**

**Supplementary tables**

Table S1 General characteristics of patients at baseline

|  | Group 1 (<10) | | | | | Group 2 (11–20) | | | | Group 3 (>20) | | | |
| --- | --- | --- | --- | --- | --- | --- | --- | --- | --- | --- | --- | --- | --- |
|  | Propofol group  (n=44) | | Control group  (n=531) | *p-*value | | | Propofol group  (n=75) | Control group  (n=481) | *p-*value | Propofol group  (n=21) | Control group  (n=35) | | *p-*value |
| Maternal age (years)  Infertility duration (years)  Maternal BMI (kg/m^2^)  Basal FSH (mIU/mL)  Basal LH (mIU/mL)  Basic E_2_ level (pg/mL)  Progesterone (ng/mL)  AFC | | 31.3 (4.7)  3.3 (2.6)  20.8 (2.4)  6.8 (2.4)  3.8 (1.7)  50.2 (18.5)  0.48 (0.2)  10.0 (5.4) | 32.8 (4.9)  3.1 (1.9)  21.7 (2.7)  6.8 (2.6)  4.3 (2.0)  51.6 (16.6)  0.53 (0.27)  10.2 (5.1) | | 0.531  0.052  0.537  0.269  0.65  0.201  0.382  0.794 | | 30.4 (4.3)  2.9 (2.3)  21.0 (3.0)  6.7 (2.8)  4.5 (1.9)  52.0 (19.4)  0.5 (0.23)  14.3 (5.2) | 31.4 (4.3)  3.1 (1.9)  21.5 (2.6)  6.6 (1.7)  4.2 (1.7)  52.2 (16.5)  0.49 (0.26)  12.2 (6.0) | 0.894  0.912  0.059  0.060  0.053  0.148  0.569  0.298 | 29.6 (4.6)  2.9 (2.5)  20.7 (3.5)  6.4 (2.1)  4.0 (1.8)  46.5 (13.9)  0.61 (0.42)  16.8 (5.6) | 30.2 (3.6)  3.0 (1.6)  21.7 (2.8)  6.0 (1.7)  4.1 (1.9)  51.0 (17.6)  0.52 (0.24)  15.0 (8.8) | 0.301  0.103  0.159  0.186  0.890  0.600  0.053  0.065 | |

Notes: Data are presented as mean (SD). No statistically significant differences were present between the two groups. BMI: body mass index; FSH: follicle stimulating hormone; LH: luteinizing hormone; E2: estradiol; AFC: antral follicle count.

Table S2 Clinical parameters

|  | Group 1 (<10) | | | | Group 2 (11–20) | | | | | Group 3 (>20) | | |
| --- | --- | --- | --- | --- | --- | --- | --- | --- | --- | --- | --- | --- |
|  | Propofol group  (n=44) | Control group  (n=531) | | *p-*value | | Propofol group  (n=75) | | Control group  (n=481) | *p-*value | Propofol group  (n=21) | Control group  (n=35) | *p-*value |
| Total does of Gn (U)  Gn duration (days)  E2 on the trigger day (pg/mL)  Pre-ovulatory follicle count | 2554.6 (717.6)  12.2 (2.0)  1672.0 (840.9)  7.4 (1.9) | | 2729.3 (980.3)  11.9 (2.9)  1521.8 (660.1)  7.3 (1.8) | 0.052  0.105  0.053  0.388 | | 2039.3 (750.5)  11.4 (2.3)  2626.1 (1278.1)  14.7 (2.8) | 2263.4 (792.5)  11.3 (2.1)  2652.1 (968.3)  14.6 (2.5) | | 0.537  0.798  0.054  0.231 | 1789.3 (805.6)  11.0 (2.0)  3142.5 (1350.9)  26.1 (5.8) | 1990.1 (843.3)  10.9 (2.2)  3044.7 (1127.0)  23.1 (2.2) | 0.495  0.254  0.290  0.002 |

Note: Data are presented as mean (SD). Gn: gonadotrophin; E2: estradiol.
